# Comparative genomic analysis and phylogeny of NAC25 gene from cultivated and wild *Coffea* species

**DOI:** 10.3389/fpls.2022.1009733

**Published:** 2022-09-16

**Authors:** Arun Kumar C. Huded, Pavankumar Jingade, Manoj Kumar Mishra, Sezai Ercisli, Gulce Ilhan, Romina Alina Marc, Dan Vodnar

**Affiliations:** ^1^Plant Biotechnology Division, Unit of Central Coffee Research Institute, Coffee Board, Mysore, Karnataka, India; ^2^Department of Horticulture, Faculty of Agriculture, Erzurum, Turkey; ^3^Food Engineering Department, Faculty of Food Science and Technology, University of Agricultural Sciences and Veterinary Medicine, Cluj-Napoca, Romania; ^4^Institute of Life Sciences, Faculty of Food Science and Technology, University of Agricultural Sciences and Veterinary Medicine, Cluj-Napoca, Romania

**Keywords:** *Coffea*, NAC25, conserved domain (NAM), sequence diversity, evolutionary relationship, expression analysis

## Abstract

Coffee is a high value agricultural commodity grown in about 80 countries. Sustainable coffee cultivation is hampered by multiple biotic and abiotic stress conditions predominantly driven by climate change. The NAC proteins are plants specific transcription factors associated with various physiological functions in plants which include cell division, secondary wall formation, formation of shoot apical meristem, leaf senescence, flowering embryo and seed development. Besides, they are also involved in biotic and abiotic stress regulation. Due to their ubiquitous influence, studies on NAC transcription factors have gained momentum in different crop plant species. In the present study, NAC25 like transcription factor was isolated and characterized from two cultivated coffee species, *Coffea arabica* and *Coffea canephora* and five Indian wild coffee species for the first time. The full-length NAC25 gene varied from 2,456 bp in *Coffea jenkinsii* to 2,493 bp in *C. arabica*. In all the seven coffee species, sequencing of the NAC25 gene revealed 3 exons and 2 introns. The NAC25 gene is characterized by a highly conserved 377 bp NAM domain (*N*-terminus) and a highly variable C terminus region. The sequence analysis revealed an average of one SNP per every 40.92 bp in the coding region and 37.7 bp in the intronic region. Further, the non-synonymous SNPs are 8-11 fold higher compared to synonymous SNPs in the non-coding and coding region of the NAC25 gene, respectively. The expression of NAC25 gene was studied in six different tissue types in *C. canephora* and higher expression levels were observed in leaf and flower tissues. Further, the relative expression of NAC25 in comparison with the GAPDH gene revealed four folds and eight folds increase in expression levels in green fruit and ripen fruit, respectively. The evolutionary relationship revealed the independent evolution of the NAC25 gene in coffee.

## Introduction

The genus *Coffea* (Family – Rubiaceae) consists of more than 125 species of which only two species *Coffea arabica* and *Coffea canephora* are commercially cultivated (Davis, 2011; Mishra and Slater, 2012). Most of the coffee species are diploid (2n = 2x = 22) and self-sterile except *C. arabica*, which is an allotetraploid (2n = 4x = 44) and self-fertile species originated by natural hybridization involving *C. canephora* and *C. eugenioides* (Lashermes et al., 1999; Pearl et al., 2004; Mishra et al., 2012). Most of the wild coffee species are native to the highlands of south-western Ethiopia to tropical African rainforest covering diverse ecological habitats of West Africa through Cameroon, Central African Republic, Democratic Republic of Congo, Uganda, Tanzania, Indian Ocean Islands, Madagascar, Comoros Islands, and the Mascarene Islands and Australasia (Davis et al., 2019; Mishra, 2019). In India, several indigenous diploid wild coffee species are discovered which include *C. bengalensis*, *C. jenkinsii*, *C. wightiana, C. khasiana* and *C. travancorensis* (Narasimhaswamy and Vishweshwara, 1963; Mishra et al., 2020; Jingade et al., 2021). Both *C. jenkinsii* and *C. khasiana* are distributed in Khasi and Jaintia Hills of the North-Eastern Himalayas and *C. bengalensis* is found growing wild in Cooch Behar and Lataguri forest of West Bengal and extended up to eastern Assam. Similarly, *C. wightiana and C. travancorensis* were wildly distributed in the forest of Travancore (Kerala) and Nilgiri hills of Tamil Nadu, India (Jingade et al., 2021). In fact, many wild crop species often offer a greater variety of traits which could form an important link between ecological adaption and the evolutionary process (Dulloo and Owadally, 1991). In this context, wild coffee species could serve as an important reservoir for the transfer and incorporate genes to breed new cultivars suitable for changed climatic conditions (Volk and Krishnan, 2020). Although plants are constantly challenged by a range of environmental stresses, they mitigate these effects by producing stress-inducing phytochemicals which involve a cascade of molecular and biochemical events. Extensive studies have underpinned that transcriptional factors (TFs) are involved in plant adaptation by acting as mediators by perceiving stress signals and directing downstream gene expression. Moreover, on an around, 7% of the coding part of the plant transcriptomes are contributed by the TFs (Singh et al., 2021). It is, therefore important to study the role of various TFs which will facilitate molecular breeding and genetic improvement of crop plants.

Among various TFs present in plants, NAC TFs constitute one of the largest groups of plant-specific transcription factors and play a very crucial role in many vital physiological and developmental processes of plants. It regulates the target genes by binding to NAC recognition sequence (NACRS) and thereby affecting different phenotypic characteristics such as lateral root development (Guo et al., 2005; Singh et al., 2021), formation and development of vegetative and floral organs (Mallory et al., 2004) and flower development (Zong et al., 2020). The NAC TFs binds to DNA or other protein kinases and regulate various physiological processes which involve secondary wall formation (Zhao et al., 2014; Hurtado et al., 2020), bud differentiation (Chen et al., 2021), embryo development (Liu et al., 2018), shoot branching (Ni et al., 2017) and fruit ripening (Martín-Pizarro et al., 2021). Among different types of NAC genes known so far, NAC25 gene play a crucial role in plants response to abiotic stress such as drought, heat, salinity and cold tolerance (Nakashima et al., 2007; Peng et al., 2009; Mao et al., 2012; Tweneboah and Oh, 2017; Han et al., 2020). It also regulates gibberellin-mediated endosperm expansion, and seed germination in plants (Sánchez-Montesino et al., 2019). In coffee, NAC TFs are known to be involved in bean development and sucrose metabolism (Dong et al., 2019).

The NAC super family protein has three distinguishable families of TFs which constitutes No Apical Meristem (NAM), Arabidopsis Transcription Activation Factors (ATAF) and the Cup-Shaped-Cotyledon families TFs (Olsen et al., 2005). The members of the NAC gene family in plants increased during evolution due to gene duplication events (Dong et al., 2019). Different NAC transcriptional factor (TF) families have been reported from different crops. About 117 different NAC TF families from Arabidopsis (Nuruzzaman et al., 2010), 151 from rice (Ooka et al., 2003), 163 from poplar (Hu et al., 2010), 79 from grape (Wang et al., 2013) and 63 from robusta coffee (Dong et al., 2019) have been identified so far. Although 63 NAC TFs are reported from *C. canephora*, the extent of molecular and sequence diversity of NAC TF across wild coffee species is unfortunately not available. Despite diploid coffee species constitute an important source for genetic variation to counter specific pests and diseases, alleviate abiotic stress, breed low caffeine coffee, and enhance organoleptic quality, there is scanty information available on the NAC gene in coffee species. The present study was therefore undertaken to explore the variability of the NAC gene among cultivated and Indian wild coffee species. Further, an attempt was also made to carry out expression analyses of the NAC25 gene in different tissues of coffee.

## Materials and methods

### Plant material

Two commercial cultivated coffee species, *viz. C. arabica* (S.795) and *C. canephora* (S.274) and five wild indigenous coffee species *viz., C. travancorensis, C. bengalensis, C. wightiana, C. jenkinsii* and *C. khasiana* belonging to different Indian geographic locations were selected in the present study. These coffee species are maintained *ex situ* at Plant tissue culture and Biotechnology Division, Coffee Research Institute, Mysore, Karnataka, India.

### Deoxyribonucleic acid extraction

The young growing leaf tips of all the seven coffee species were collected and total genomic DNA was isolated using the modified CTAB protocol described by Mishra et al. (2020). The DNA quality was tested by separating it on 0.8% agarose gel stained with ethidium bromide (0.5 μg/ml) and quantified using Nanodrop (Eppendorf). The DNA samples were diluted to a working concentration of 10 ng/μl and stored at −20°C for future uses.

### Ribonucleic acid extraction

The total RNA was extracted from the six different tissues viz., root, young leaf, immature flower bud, flower, green fruit and ripen fruit of *C. canephora* (S.274) using the potassium acetate method described by Huded et al. (2018). The total RNA was quantified using Nanodrop (Thermo Fisher) at 260/280 nm and was reverse transcribed into cDNA using a high-capacity RNA to cDNA conversion kit (Applied Biosystem, USA). Further, the first-strand cDNA synthesis was carried out in 20 μl reaction volume containing 10 μl of 2 × RT buffer, 1.0 μl of 2 × enzyme mixes, 4.0 μl of RNA sample and 5.0 μl of nuclease-free water. Reverse transcription was performed using a thermal cycler (BioRad) at 37°C for 60 min and 95°C for 5 min. and the cDNA was stored at −80°C for future use.

### Primer designing and polymerase chain reaction amplification

The NAC25 like transcript gene sequence of *Coffea eugenioides* (XM_027296760.1; XM_027296758.1 and XM_027296751.1) available in the NCBI GenBank database^1^, was downloaded and primer pairs were designed using Primer3Plus program^2^. The designed primer pairs were checked byPrimer-BLAST tool in NCBI database^3^. The PCR amplification was carried out with genomic DNA samples of seven coffee species in a reaction volume of 15 μl using Bio-Rad Thermal cycler S1000. PCR reaction mixtures contained 3.0 μl of DNA (10 ng/μl) (cDNA samples as a template for characterization of transcript), 3.0 μl each of 3 μM primer (Forward primer – 5′-CGCAAATAGAGGCCTCAGCC-3′ and Reverse primer – 5′-CGCATGGCTCCCAAGATTCT-3), 1.5 μl of 2 mM dNTPs, 1.5 μl of 10X Taq buffer, 1.5 μl of 25 mM MgCl_2_ (Thermo Fisher Scientific, Waltham, USA) and 0.3 μl of 3 units/μl Taq DNA polymerase enzyme (GeNei, Bangalore, India). The reaction volume was made up to 15 μl using sterile distilled water. Standard PCR cycling parameters were used which includes an initial denaturation step of 5 min at 94°C, followed by 30 cycles of 94°C for 30 s, Primer annealing at 62°C for 1 min, primer extension at 72°C for 2 min and final extension of 10 min at 72°C. The amplified PCR products were mixed with 5 μl Bromophenol blue dye (99.5% deionized formamide, 10 mM EDTA pH 8, 0.05% Bromophenol blue, xylene-cyanol dye solution, 1 μl pure sterile water) and separated on 1.5% agarose gel (SeaKem, Rockland USA) containing 0.5 μg ethidium bromide/ml in 1x TBE (Tris-HCl, Boric acid, EDTA) buffer. After electrophoresis, the gels were visualized and documented using Gel Doc System (BioRad) with Multi Analyst software program.

### Cloning and sequencing

The purified PCR products of NAC gene ∼2.4kb were ligated into pGEM-T easy cloning vector as per manufacturer’s instructions (pGEM-T clone kit, Promega) and transformed into *Escherchia coli* DH5α competent cells using the heat shock method. Successful transformants were selected using blue/white screening and colony PCR. The colonies tested positive for PCR were cultured and the recombinant plasmids were extracted from the culture using the alkaline lysis method as described by Sambrook and Russell (2001). The isolated plasmids were sequenced by the commercial sequencing facility at Eurofins Genomics India Pvt. Ltd., Bangalore, Karnataka, India.

### Sequence analysis and construction of phylogenetic tree

The full-length NAC gene sequences of seven coffee species obtained by sequencing were BLASTn searched against the NCBI database (see text footnote 1). Based on the sequence homology studies, the intronic and exonic regions were identified from the full-length NAC gene sequence. Further, by joining the exonic regions full-length transcript sequences were derived and translated to protein sequence using the EMBOSS-Transeq tool of EMBL-EBI. Successively, the homologies of these protein sequences were analyzed using the BLASTp tool of the NCBI database. Further, to access the extent of variation in NAC25 gene sequences of different coffee species, multiple sequence alignment was performed using the MUSCLE program of MEGA X version 10.2.4 software with the default parameter (Kumar et al., 2018). The phylogenetic tree was constructed using full-length NAC25 gene sequences, by following the maximum likelihood (ML) method, based on the Tamura-Nei model (Tamura and Nei, 1993) with 1,000 bootstrap replications in MEGA X Version 10.2.4 Software developed by Penn State University, USA. Consequently, the protein sequences were analyzed and a phylogenetic tree was constructed using the maximum likelihood (ML) method based on the Jones-Taylor-Thornton (JTT) model (Jones et al., 1992) with 1,000 bootstrap replications in MEGA X Version 10.2.4.

Moreover, to annotate the NAC genes obtained from seven coffee species, a phylogenetic tree was constructed using these protein sequences along with a total of 73 protein sequences belonging to 37 different NAC genes of *C. eugenioides* retrieved from the NCBI database and 63 protein sequences of different NAC genes identified by Dong et al. (2019). Further, to ascertain the evolutionary relationship of NAC25 genes, a global phylogenetic analysis was carried out using the 147 NAC25 like protein sequences belonging to 72 different crop species retrieved from the NCBI protein database. Both the phylogenetic tree was constructed using the maximum likelihood (ML) method based on the Jones-Taylor-Thornton (JTT) model (Jones et al., 1992) with 1,000 bootstrap replications in MEGA X Version 10.2.4.

### Sequence characterization

In the present study, the full length NAC gene was isolated from *C. canephora* (S.274) which is a diploid cultivated species with commercial importance and the same was considered as the reference sequence. The variations in the NAC gene sequence among six coffee species were analyzed and compared with *C. canephora* sequence. The sequence variations were studied using the multiple sequence alignment (MSA) tool of BioEdit software version 7.2.5 (Hall, 1999). Also, using multiple sequence alignment, Single Nucleotide Polymorphism (SNP), Insertion-Deletions (InDels) were manually identified. Further, the species-specific InDels sequences were identified for six coffee species by considering *C. canephora* (S.274) as a reference sequence. Further, the nucleotide base composition of the NAC gene from all the seven coffee species was determined using the BioEdit software version 7.2.5 (Hall, 1999). In addition, the NAC protein sequences from all the coffee species were analyzed and the amino acid content along with the theoretical isoelectric point (pI) was determined using the ExPASy-ProtParam online tool (Gasteiger et al., 2005). The hydrophilic coefficient was determined using ExPASy-ProtScale online tool (Kyte and Doolittle, 1982). Moreover, the conserved NAM domain was identified from full-length NAC protein sequences obtained from seven coffee species using conserved domain database (CDD) version3.18-55570 PSSMs of NCBI^4^ with default parameters. In full- length NAC25 gene (2.4kb) of all seven coffee species, exons and introns were marked using Gene Structure Display Server, version 2.0 (GSDS2.0) (Hu et al., 2015) and the NAM conserved domain was marked manually for easy distinction.

### Expression analysis

To investigate the site of expression of the NAC gene, six different tissues viz., root, leaf, immature flower bud, flower, green fruit and ripen fruit of *C. canephora* (S.274) were selected for RT-PCR assay. The RT-PCR assay was performed using the Bio-Rad CFX-96 real-time system. The cDNA samples of six tissues were quantified using Nanodrop (Thermo Fisher) and diluted to uniform concentration for expression studies. Two replicates of each tissue in the PCR reaction mixtures were confined by the 1.2 μl of cDNA samples as a template, 1.5 μl each of 3 μM forward primer and reverse primer and 10.0 μl of SYBER green master mix (Thermo Fischer). The reaction volume was made up to 20 μl using sterile distilled water. The forward (5′-AAAGCTCGGCAAAGGAATGA-3′) and reverse primer (5′-CCCACASCTCACCATCAATGC-3′) pair amplifying 425bp from the exonic region of the highly variable C-terminal of NAC25 gene was designed and used for expression studies. The GAPDH specific gene (Forward primer –5′-ATTGTTGAGGGCCTTATGACCACT-3′ and Reverse primer – 5′-TGCCCGCAGCCTCCTCCTTA-3′) was used and the expression data was analyzed using 2^–Δ^ ^Δ^ *^Ct^* method. The GAPDH gene was used as a reference gene.

## Results and discussion

### Polymerase chain reaction Amplification, cloning and sequencing

**FIGURE 1:**
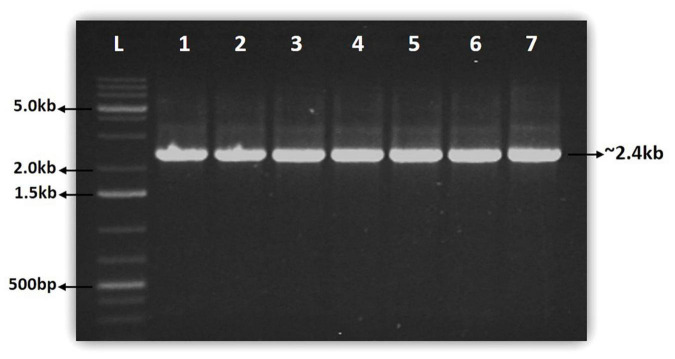
Gel pictures showing the amplification of full length NAC gene from seven coffee species. **Lane L:**1kb plus GeneRuler from Thermo Fischer Scientific, **Lane 1:** *C. canephora* (S.274), **Lane 2:** *C. arabica* (S.795), **Lane 3:** *C. travancorensis***, Lane 4:** *C. bengalensis* **Lane 5:** *C. wightiana* **Lane 6:** *C. jenkinsii* **Lane 7:** *C. khasiana*.

The full-length NAC gene of 2.4 kb was amplified from the genomic DNA samples of seven coffee species using NAC gene-specific primer (Figure 1). In each species, the 2.4 kb single amplicon was cloned using the pGEM-T easy cloning vector cloning kit and sequenced. The sequencing of the NAC gene amplicons yielded a minimum of 2.4 kb sequence in all the seven coffee species (Table 1). The highest NAC gene sequence of 2,493 bp was found in *C. arabica* (S.795) whereas the lowest sequence size of 2,456 bp was obtained in *C. jenkinsii* (Table 1).

**TABLE 1:** Table depicting the sequence size and nucleotide base contents of the NAC25 gene isolated from *Coffea* species.

Samples	Base pair		Total base pairs	Nucleotide content (%)				G + C content (%)	Accession number
	Exonic region	Intronic region		A	C	G	T		
***I***	1,395	1,073	2,468	666 (26.99)	499 (20.20)	454 (18.40)	849 (34.40)	38.60	OL627349
***II***	1,395	1,098	2,493	674 (27.04)	502 (20.14)	456 (18.29)	861 (34.54)	38.43	OL627350
***III***	1,386	1,102	2,488	685 (27.53)	509 (20.46)	459 (18.45)	835 (33.56)	38.91	OL627351
***IV***	1,386	1,095	2,481	673 (27.13)	497 (20.03)	461 (18.58)	850 (34.26)	38.61	OL627355
***V***	1,401	1,089	2,490	680 (27.31)	500 (20.08)	463 (18.59)	847 (34.02)	38.67	OL627352
***VI***	1,386	1,070	2,456	665 (27.08)	492 (20.03)	452 (18.40)	847 (34.49)	38.44	OL627354
***VII***	1,395	1,073	2,468	659 (26.70)	509 (20.62)	459 (18.60)	841 (34.08)	39.22	OL627353
**Average**	**1,392.0**	**1,085.7**	**2,477.7**	**671.71 (27.09)**	**501.14 (20.22)**	**457.71 (18.47)**	**847.14 (34.19)**	**38.72**	

***I -** C. canephora (S.274)**; II-**C. arabica (S.795)**; III -** C. travancorensis**; IV -** C. bengalensis**; V -** C. wightiana**; VI -** C. jenkinsii**; VII -** C. khasiana.*

### Sequence similarity and phylogeny based gene annotation

The sequence homology of the seven full-length NAC gene sequences was verified using BLASTn tool of the NCBI database. The BLAST analysis resulted in 99.06 to 100% sequence similarity with NAC25 like transcript sequence of *C. eugenioides* (XM_027296760.1, XM_027296758.1 and XM_027296751.1) with query coverage of 46 to 53%. The lower percentage of query coverage was due to the absence of full- length NAC gene (exon + intron) sequences in the NCBI database. However, the higher sequence similarity can be attributed to the presence of the NAC25 transcript sequences in the NCBI database.

**FIGURE 2:**
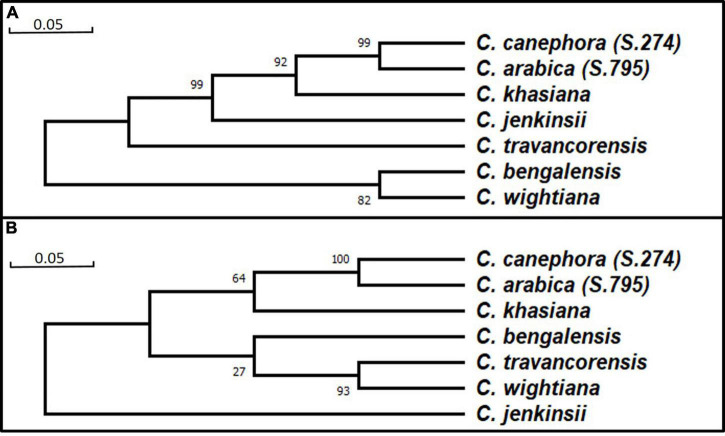
Phylogenetic tree constructed usingFull length nucleotide sequence by Maximum Likelihood approach using Tamura-Nei model **(A)** and Protein sequence by Maximum Likelihood approach Jones-Taylor-Thornton (JTT) model **(B)**.

Based on homology studies, the NAC25 transcript sequences of seven coffee species were derived from their respective full-length NAC25 sequences by delimiting the intronic regions. These transcript sequences were converted to protein sequences and the amino acid sequence homology was studied. These homology studies revealed the highest sequence homology of 84.80 to 91.16% with *C. eugenioides* NAC25 (XP_027152552.1) with 99% query coverage. Further, the phylogenetic trees were constructed using both full- length nucleotide and the protein sequences of seven coffee species (Figure 2). The clustering pattern of the seven coffee species in both the phylogenetic trees was largely similar. The seven coffee species are divided into two main clusters (Figures 2A,B). *C. arabica* (S.795) and *C. canephora* (S.274) were clustered together and closely placed along with *C. khasiana*, whereas, *C. bengalensis* and *C. wightiana* were closely placed with *C. travancorensis* in the phylogenetic tree constructed using full length and protein sequences. However, the *C. jenkinsii*, which was placed close to *C. khasiana* in the phylogenetic tree constructed using full-length NAC25 gene sequence, formed its own cluster in the tree constructed using protein sequences (Figure 2B). The change in the clustering pattern of *C. jenkinsii* could be attributed to the variations in the NAC25 gene sequence at the transcript level. Since, NAC TFs are associated with stress responses in plants, the evolutionary changes occurred in NAC gene sequence of *C*. *jenkinsii* could be attributed to the species adaptive mechanism in the course of evolution. The *C. jenkinsii* transcript sequence had 17 (1.22%) non-synonymous single nucleotide polymorphisms (nsSNP) coupled with 9bp deletions while *C. khasiana* transcript sequence had 57 (4.08%) nsSNPs with no deletions in comparison to the reference sequence.

**FIGURE 3:**
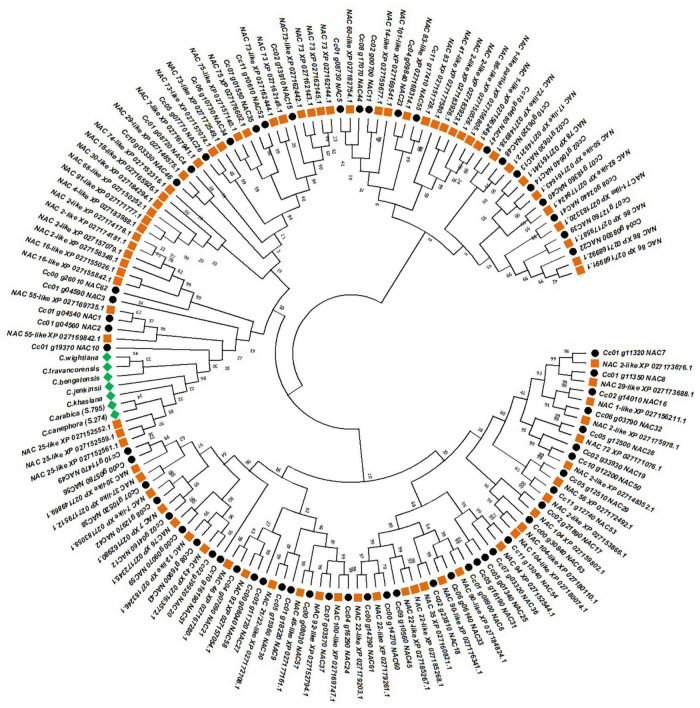
Phylogenetic tree constructed by using 81 different classes of NAC protein sequences of *C. eugenioides* (orange squares), 63 different classes of NAC protein sequences of *C. canephora* (black circle) and the NAC protein sequences isolated in the present study (green square) using Maximum Likelihood approach JTT-model.

The phylogenetic tree constructed, to annotate the seven NAC genes isolated in the present study, classified the 151 (including our seven NAC genes) different types of NAC genes into two major clusters (Figure 3). The first major cluster is composed of 33 NAC genes of different types of which 20 belong to *C. eugenioides* and 13 from *C. canephora* (Figure 3). The second major cluster was subdivided into two minor clusters and each minor cluster is further subdivided into two sub minor clusters. The NAC genes isolated from seven coffee species were closely grouped together with three NAC25 like genes of *C. eugenioides* in one of the sub minor clusters (Figure 3). Further, the NAC10 of *C. canephora* were also placed in the same sub minor cluster. However, the NAC10 gene of *C. canephora* had a short peptide length and only one intron unlike the NAC genes isolated in the present study which had an average peptide length of 463 amino acids and two introns. The NAC25 like genes of *C. eugenioides* had the peptide length of 432 amino acids and shared 99% sequence homology with the NAC gene isolated from different coffee species in the present study. Hence, all the seven NAC genes isolated from different coffee species were characterized as NAC25 genes further, these sequences were submitted to the BankIt portal of NCBI and accession numbers were obtained (Table 1).

**FIGURE 4:**
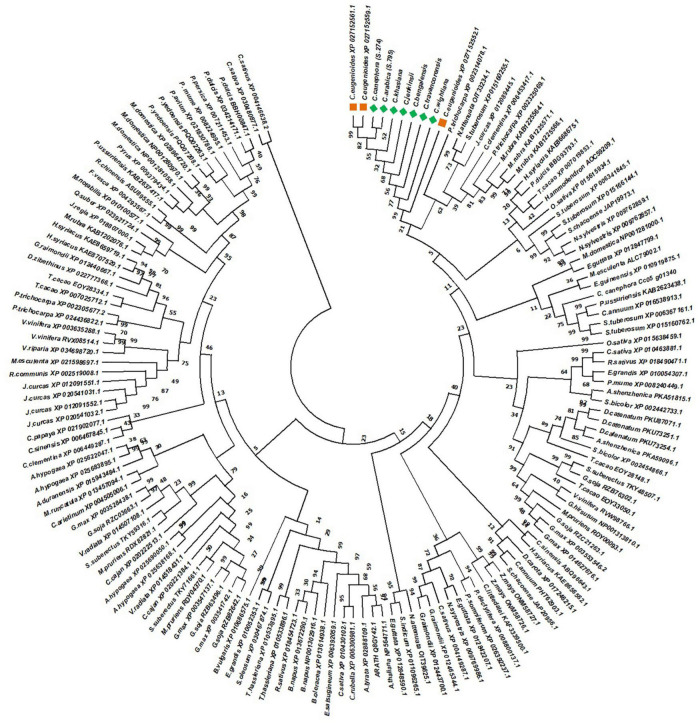
Phylogenetic tree constructed by using 147 NAC25 protein sequences belonging to 72 different crop species along with NAC25 protein sequences of *C. eugenioides* (orange squares) and NAC protein sequences isolated in the present study (green square) by Maximum Likelihood approach Jones-Taylor-Thornton (JTT) model.

The evolutionary relationship of NAC25 genes isolated in the present study was further elucidated by constructing a separate phylogenetic tree (Figure 4) comprising 147 NAC25 protein sequences belonging to 72 different crop species (Supplementary Table 1). The phylogenetic tree revealed a close relationship between the NAC25 sequences, obtained from seven coffee species with the NAC25 sequence of *C. eugenioides*. Further, the NAC25 like genes isolated from different *Coffea* species were closely clustered with NAC25 likes genes of two *Solanum* species such as *Nicotiana attenuata* and *Solanum tuberosum*. The close clustering of *Solanum* NAC25 like genes with NAC25 like genes of *Coffea* is concurrent with an earlier study made by Lin et al. (2005) which suggested that compared to Arabidopsis, coffee is closely related to Solanaceae and tomato a member of Solanaceae had a nearly perfect gene-for-gene match with coffee and could have similar functions. Further, coffee and tomato have a similar genome size, chromosome karyotype (tomato, *n* = 12; coffee n = 11), and chromosome architecture and both belong to the Asterid I clade of dicot plant families Lin et al. (2005).

### Sequence characterization

The full-length sequence of the NAC25 gene, isolated from the seven coffee species ranged from 2,456 bp (*C. jenkinsii*) to 2,493 bp (*C. arabica*) with an average length of ∼2,477 bp (Table 1). Considerable variation in the NAC25 gene sequence length was reported from different crops plant species. In *Arabidopsis thaliana*, the length of the NAC25 gene (gene id: AT1G61110.0) was 1,619 bp in size (Alvarado and Terry, 2005), whereas, the length of the NAC25 gene isolated from *Malus baccata* was 1,122 bp (Han et al., 2020) and the gene isolated from *Malus domestica* (Li et al., 2020) was 1,335 bp. The average length of the NAC25 gene isolated from coffee species was higher when compared to the NAC25 genes isolated from different crop species. Further, characterization of the NAC25 gene revealed that the NAC25 genes isolated from the seven coffee species were composed of three exons spliced by two introns. Our finding is in concordance with earlier studies by Alvarado and Terry (2005) on the characterization of the NAC25 gene in *A. thaliana* and Dong et al. (2019) on the NAC25 gene in *C. canephora*. The average exon size of ∼1,392 bp and average intron size of ∼1,073 bp were obtained from the NAC25 gene of seven coffee species (Table 1).

In the present study, the peptide length of the NAC25 gene was ranged from 461 (*C. travancorensis*, *C. bengalensis* and C. *jenkinsii*) to 466 (*C. wightiana*) with an average of 463 amino acids and the hydrophilic coefficient was −0.490 (Table 2). Interestingly, the peptide length of NAC25 isolated from cultivars *C. arabica* and *C. canephora* was 464 amino acids. The peptide length of NAC25 gene isolated from *C. canephora* in the present study was higher than the length of *CocNAC25* gene reported by Dong et al. (2019). This disparity in lengths could be attributed to the classification and nomenclature of NAC genes adopted by Dong et al. (2019). Dong et al. (2019) categorized the NAC genes based on their positions on chromosomes unlike, the NAC genes identified in the present study, which were classified based on sequence homology studies with the annotated NAC25 gene sequences available in the public database. However, the *CocNAC36* and *CocNAC43* genes identified by Dong et al. (2019) had peptide lengths of 400 and 455 amino acids, respectively. These lengths correspond to the peptide length of NAC25 genes isolated in the present study. Further, the variations in peptide length among the NAC25 genes isolated from different crops were observed. The peptide length of *ANAC25* isolated from *A. thaliana* was 323 amino acids (Alvarado and Terry, 2005), the length of *FtNAC25* isolated from *Fagopyrumtataricum* was 436 amino acids (Liu et al., 2019) and the length of *MbNAC25* isolated from *Malus baccata* was 373 amino acids (Han et al., 2020). Further, the peptide sequence of NAC25 protein revealed the presence of amino acids such as aspartic acid, glycine, leucine, proline, serine and asparagine in larger numbers, unlike methionine, tryptophan and cysteine which were found in relatively lower numbers (Table 2). The earlier study by Han et al. (2020) also reported that the presence of leucine, lysine, proline, serine and threonine in higher numbers.

**TABLE 2:** Table depicting the assessment of amino acids of NAC25 gene isolated from *Coffea* species.

		*I*	*II*	*III*	*IV*	*V*	*VI*	*VII*	Average
**Amino Acid**		464	464	461	461	466	461	464	**463**
**Mol. Weight (kDa)**		51.80	51.83	51.46	51.61	52.35	51.47	51.96	**51.78**
**Isoelectric point, pI**		5.74	5.74	5.97	5.86	6.13	6.00	5.60	**5.86**
**Highest Amino Acid (%)**	**Asp**	8.19	8.19	8.24	8.02	8.15	8.02	8.83	**8.23**
	**Gly**	7.32	7.32	6.94	6.72	6.65	7.15	7.75	**7.12**
	**Leu**	6.68	6.89	6.29	6.50	6.65	6.29	6.03	**6.48**
	**Pro**	9.05	9.05	10.62	9.54	9.65	9.11	9.91	**9.56**
	**Ser**	8.83	8.62	7.80	8.67	8.15	8.67	7.54	**8.33**
	**Asn**	7.75	7.75	7.59	7.80	7.51	8.02	7.32	**7.68**
**Lowest Amino Acid (%)**	**Met**	2.15	2.15	1.95	2.16	2.14	2.16	2.37	**2.15**
	**Trp**	0.64	0.64	0.65	0.65	0.85	0.65	0.65	**0.68**
	**Cys**	1.29	1.29	1.30	1.30	1.28	1.30	1.29	**1.29**
**Hydrophilic coefficient**		**−**0.490	**−**0.490	**−**0.490	**−**0.490	**−**0.490	**−**0.490	**−**0.490	**−0.490**

***I -** C. canephora (S.274)**; II-**C. arabica (S.795)**; III -** C. travancorensis**; IV -** C. bengalensis**; V -** C. wightiana**; VI -** C. jenkinsii**; VII -** C. khasiana.*

Also, in the present study, the NAC25 protein had the molecular weight ranged from 51.46 KDa (*C. travancorensis*) to 52.35KDa (*C. wightiana*) with an average of 51.78KDa and the theoretical isoelectric point (pI) ranged from 5.60 (*C. khasiana*) to 6.13 (*C. wightiana*) with an average of 5.86 (Table 2). In a previous study, the molecular weight of NAC25 genes isolated from different crops such as *A. thaliana* (Alvarado and Terry, 2005), *C. canephora* (Dong et al., 2019), *F. tataricum* (Liu et al., 2019), *M. baccata* (Han et al., 2020) and *M. domestica* (Li et al., 2020) were 36.31KDa, 34.27KDa, 48.62KDa, 41.49KDa and 43.189 KDa, respectively and their respective theoretical isoelectric point was 8.1, 8.9, 4.75, 8.7, and 7.02.

### Conserved domain

**FIGURE 5:**
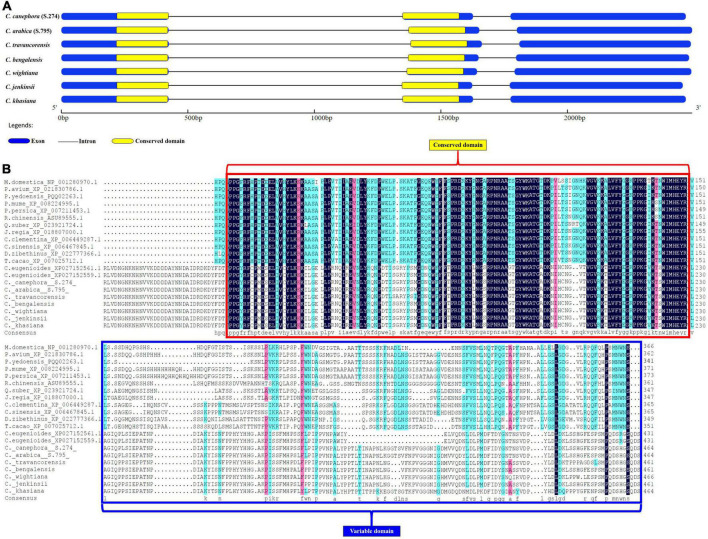
**(A)** Figure depicting the exon, intron and conserved domain of full length NAC25 gene isolated from seven coffee species. **(B)** Comparison of NAM conserved domain of NAC25 gene with other crop species.

The NAC gene sequences isolated from seven different coffee species had the typical characteristics of the NAC gene family. Proteins of the NAC family have the characteristic NAC domain of about 150 amino acids at the N-terminus region (NAM domain) and area highly conserved region (Mohanta et al., 2020). In contrast, the large C-terminal regions of NAC proteins are highly divergent and act as a transcriptional activator or repressor and therefore known as functional domain (Tran et al., 2004; Hu et al., 2006; Kim et al., 2007; Bian et al., 2021). The N-terminus of all the NAC25 genes had the presence of a highly conserved domain (NAM domain) and the variable C-terminus region carrying transcriptional regulatory sites. In the present study, the length of the conserved NAM domain was uniform in all the species with 377bp conserved sequence length (125 amino acids) (Figure 5). However, variation in the position of conserved domain across the species was observed (Figure 5A). The analysis of the conserved domain using the online Hidden Markov Model (HMM) program showed the best and maximum hit with the NAM domain of pfam02365 (PFAM ID: 02365). The analysis of the peptide sequence of NAM domain from all the seven coffee species revealed a higher content of glycine followed by lysine, proline and arginine. This NAM domain plays an important role in maintaining the structure and function of NAC proteins. Hence, the conserved nature of NAM domain was confirmed through multiple sequence alignment using NAC25 protein sequences of 10 coffee species and 12 different crop species (Figure 5B). The analysis demonstrated the evolutionarily conserved nature of the NAM domain across the species. Unlike the NAM domain, the highly variable region at the upstream region is known as the C-terminal region which is also said to be the function domain of NAC proteins.

### Sequence variation studies

#### Single nucleotide polymorphism/polymorphic sites

To study the sequence variations in NAC25 genes isolated from seven coffee species, the full-length NAC25 gene sequences of individual species were aligned with the reference sequence (*C. canephora*) and the SNPs were identified. The analysis revealed that *C. travancorensis* had the highest number of SNPs (142) unlike *C. arabica* which had the lowest number of SNPs (6) (Table 3 and Supplementary Table 1). Further, the total number of SNPs in exonic and intronic regions of the NAC25 gene isolated from each species was computed and presented in Table 3. It was observed that the frequency of detected SNPs ranged from 0.24% (*C. arabica*) to 5.75% (*C. travancorensis*). The sequencing of the NAC25 gene also revealed that an average of one SNP per every 40.92bp in the coding region and 37.7bp in the intronic region with an average of one SNP per 39bp of the entire gene length. Further, the total SNPs in exonic and intronic were classified into synonymous (sSNPs) and non-synonymous SNPs (nsSNPs) and it was observed that the nsSNPs are 8-11 fold higher compared to sSNPs in the non-coding and coding region of the NAC25 gene, respectively.

**TABLE 3:** Table depicting the sequence polymorphism and diversity of the NAC25 gene with reference to *Coffea canephora* (S.274).

				*C. arabica* (S.795)	*C. travancorensis*	*C. bengalensis*	*C. wightiana*	*C. jenkinsii*	*C. khasiana*
**Total alignment length (bp)**				**2,496**	**2,516**	**2,506**	**2,519**	**2,468**	**2,473**
**Total number of monomorphic sites (bp)**				**2,459**	**2,298**	**2,400**	**2,362**	**2,433**	**2,377**
**Identified Single Nucleotide Polymorphisms (SNPs) at**	**Exonic region**	**s (%)**		–	5 (0.36)	–	4 (0.28)	2 (0.14)	6 (0.43)
		**ns (%)**		1 (0.07)	56 (4.01)	15 (1.07)	41 (2.94)	17 (1.22)	57 (4.08)
	**Intronic region**	**s (%)**		1 (0.09)	5 (0.46)	4 (0.37)	4 (0.37)	2 (0.19)	3 (0.28)
		**ns (%)**		4 (0.37)	76 (7.08)	24 (2.23)	28 (2.60)	2 (0.19)	20 (1.86)
**Total number of polymorphic sites in bp(%)**				**6 (0.24)**	**142 (5.75)**	**43 (1.74)**	**77 (3.12)**	**23 (0.93)**	**86 (3.48)**
**Identified Insertions and Deletions (InDels)** **at**	**Exonic region**	**F**	**In (%)**	–	–	–	–	–	–
			**Del (%)**	–	–	–	3 (0.21)	–	–
		**NF**	**In (%)**	–	–	–	18 (1.29)	–	–
			**Del (%)**	–	9 (0.64)	9 (0.64)	9 (0.64)	9 (0.64)	–
	**Intronic region**	**F**	**In (%)**	28 (2.61)	18 (1.68)	11 (1.02)	6 (0.56)	–	5 (0.46)
			**Del (%)**	3 (0.28)	16 (1.49)	13 (1.21)	14 (1.30)	3 (0.28)	5 (0.46)
		**NF**	**In (%)**	–	30 (2.79)	27 (2.51)	27 (2.51)	–	–
			**Del (%)**	–	3 (0.28)	3 (0.28)	3 (0.28)	–	–
**Total number of mutations in bp (%)**				**31** (1.25)	**76** (3.08)	**63** (2.55)	**80** (3.24)	**12** (0.48)	**10** (0.40)

**s**- Synonymous; **ns-** Non-synonymous; **F-** Frameshift mutation; **NF-** Non-frameshift mutation; **In-** Insertion; **Del-** Deletion.

In this study, the level of polymorphism created by the frequency of SNP is comparable to that reported by Kolkman et al. (2007) who reported an average of one SNP every 45.7 bp in sunflower but higher than what was previously reported for grapevine by Riahi et al. (2013) which recorded an average of one SNP per every 63 bp in the NAC gene sequence. The frequency of SNP in gene sequence varies considerably in different species. For example, in maize, there is one SNP for every 100 bp (Rafalski, 2002) whereas an average of 1 SNP per every 77 bp was observed for cotton (An et al., 2008) and one SNP per 273 bp was detected in soybean (Zhu et al., 2003). In an earlier study, Mishra et al. (2011) reported an average of one SNP for every 47 bp in the EST sequence of various coffee species and the present findings are comparable with the earlier results. In the present study, the frequency of SNP in the non-coding region is slightly higher than the coding region which is in agreement with the earlier studies showing that the mutations carried out with a higher frequency in non-coding regions than in coding regions (Kolkman et al., 2007; An et al., 2008). The presence of an eightfold higher frequency of nsSNPs over sSNPs in the NAC25 gene resulted in the change of the encoded amino acid in various coffee species, although no drastic change in the amino acid coding was recorded between two cultivated species. This could be important because SNPs play a significant role in the evolution of candidate genes coding for functional traits in plants. Indeed, most of the Indian coffee species have low/trace caffeine in their fruits compared to the cultivated varieties and therefore further work is needed to establish whether NAC25 gene has any role in controlling the caffeine metabolism.

#### Insertions and deletions

Insertions and deletions (InDels) are the results of one or more mutation processes, including DNA impairing (Levinson and Gutman, 1987), transposition (Bennett, 2004) crossover (Wang et al., 2018) and/or slippage (Moghaddam et al., 2014). Considering the importance, the InDels in the NAC25 gene sequence of each species were analyzed by aligning the individual gene sequence with the NAC25 sequence of *C. canephora* (S.274). The comparative analysis revealed that, *C. khasiana* had the lowest number of InDels and *C. wightiana* had the highest number of InDels (Table 3). The highest number of insertions, as well as the highest number of deletions, was observed in *C. wightiana* (51 insertions and 29 deletions) and no insertions were found in *C. jenkinsii*. The NAC25 gene from *C. arabica* had the lowest number of deletions. Even though InDels affect the sequence diversity, they are considered to be relatively rare events when compared to point mutations (Pascarella and Argos, 1992; Kondrashov, 2003). Unlike substitutions, InDels are less likely to be selectively neutral under constant selective pressure and are frequently deleterious (Panchenko et al., 2005). Further, the InDels that are accumulated during evolution may not be deleterious to a species or a group of species but, they can change protein structures and function (Jiang and Blouin, 2007; Wolf et al., 2007; Cai et al., 2008) leading to adaptations to new environments (Grishin, 2001). Considering the diverse role of NAC TFs in species adaption and stress resistance, it will be interesting to explore whether the structural variability (InDels and SNPs) of NAC25 gene obtained in different coffee species could be attributed to the functional significance.

#### Identification of species specific insertion-deletion

Sequence diversity was observed in the NAC25 gene isolated from seven coffee species. Hence, an attempt was made to identify species-7specific InDels in the NAC25 gene sequence by considering the NAC25 sequence of *C. canephora* diploid cultivated species as a reference. The comparative analysis fetched the sequence-specific InDels in all the species (Table 4). The highest number of species-specific insertions was found in the intronic region of all the species except *C. jenkinsii* (Table 4). None of the species had the presence of specific insertions in the exonic region except *C. wightiana* which had the insertion of 18 bp in the exonic region (Table 4). At the intronic region of the NAC25 gene, the lengths of species-specific insertion ranged from 24 bp in *C. travancorensis*, *C. bengalensis* and *C. wightiana* to 28 bp in *C. arabica* (Table 4). Similarly, at the exonic region of the NAC25 gene, the species-specific deletions of 9bp were observed in all the species except *C. arabica* and *C. khasiana*. At the intronic region of the NAC25 gene, *C. travancorensis*, *C. bengalensis* and *C. wightiana* had the specific deletion of 11bp (Table 4).

**TABLE 4:** Table depicting species specific Insertions and Deletions (InDels) causing frameshift (F) and non-frameshift (NF) mutation at the NAC25 gene sequence of six coffee species with reference to NAC25 gene of *Coffea canephora* (S.274).

Sr. no.	Species	Position	bp	INSERTION	DELETION	Total
1	***C. arabica***	692	1		C	**31**
		714	1		C	
		1,152-1,179	28	TACTATATATTGGT GTAATTAACCCTTC		
		1,211	1		T	
2	***C. travancorensis***	427-432	5	AGAACC		**76**
		438-464	11	CCTATTTAAAA		
		637-641	5	TTTAG		
		663	1	G		
		713	1		C	
		735	1		C	
		923-933	11		TTAGTTCTCCG	
		1,061-1,063	3		ACT	
		1,175-1,198	24	TACTATATATTGG TGTAACCCTTC		
		1,236	1	T		
		1,276	1		G	
		1,761-1,762	2		TA	
		2,315-2,323	9		GGGTACATC	
3	***C. bengalensis***	690	1		C	**63**
		712	1		C	
		869-872	4	TATA		
		904-914	11		TTAGTTCTCCG	
		1,042-1,044	3		TAC	
		1,156-1,179	24	TACTATATATTGGT GTAACCCTTC		
		1,210-1,213	4	GCTT		
		1,221	1	T		
		1,267-1,269	3	AAA		
		1,322-1,223	2	TA		
		2,305-2,313	9		GGGTACATC	
4	***C. wightiana***	690	1		C	**80**
		712	1		C	
		869-872	4	TATA		
		904-914	11		TTAGTTCTCCG	
		1,042-1,044	3		ACT	
		1,156-1,179	24	TACTATATATTGG TGTAACCCTTC		
		1,212	1		G	
		1,262-1,264	3	AAA		
		1,317-1,318	2	TA		
		1,904-1,921	18	TCGGAATTCT GATCATCA		
		2,318-2,326	9		GGGTACATC	
		2,456	1		C	
		2,462	1		G	
		2,471	1		T	
5	***C. jenkinsii***	690	1		C	**12**
		712	1		C	
		1,183	1		T	
		2,267-2,275	9		GGGTACATC	
6	***C. khasiana***	690	1		C	**10**
		806	1	G		
		1,636-1,639	4	GGTA		
		1,668	1		T	
		1,675	1		G	
		1,698-1,699	2		AA	

### Expression studies

**FIGURE 6:**
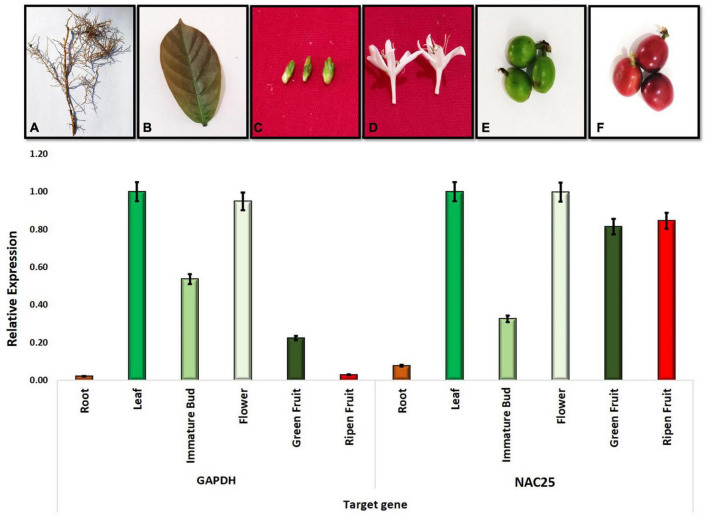
Figure depicting the relative expression of NAC25 gene in different tissues of coffee **(A)** Root; **(B)** Leaf; **(C)** Immature Bud; **(D)** Flower; **(E)** Green fruit and **(F)** Ripen fruit.

A preliminary study was carried out to understand the relative level of expression of the NAC25 gene in *C. canephora* (S.274) using six different tissues such as root, leaf, immature flower bud, flower, green fruit and ripen fruit was studied using GAPDH as a reference gene. All the six tissues showed the expression of the NAC25 gene however, the expression levels are varied (Figure 6). Among the six tissues studied, higher expression levels were observed in leaf and flower tissues. The lowest expression was observed in root tissue followed by immature bud. Further, the relative expression of NAC25 in comparison with the GAPDH gene revealed four folds and eight folds increase in expression levels of the NAC25 gene in green fruit and ripen fruit, respectively (Figure 6). The higher relative expression levels of NAC25 in fruit tissue obtained in the present study is in concordance with the results obtained by Dong et al. (2019). Dong et al. (2019) carried out the expression analysis of 63 different NAC genes in four different stages of fruits such as small green, large green, yellow and red fruit of *C. canephora* and observed that 54 out of 63 different types of NAC genes showed different levels of expression during different stages of fruit development. Sánchez-Montesino et al. (2019) also obtained higher expression of NAC gene during endosperm development. Further, Han et al. (2020) observed higher level of NAC25 expression in young leaves and stem of *Malus baccata* and opined that the higher level of expression of NAC25 in young levees could be associated with abiotic stress tolerance. In Solanaceae family, NAC TFs confer resistance to fungal infection, drought and salt stress (Singh et al., 2013; Zhu et al., 2014). Since the NAC25 transcription factor isolated from coffee showed a close relationship with the NAC TF of members of the Solanaceae family, it could be possible that they could have similar functions.

## Conclusion

In most coffee-growing countries, sustainable coffee cultivation is under considerable threat due to the increased prevalence of biotic and abiotic stress escalated by global climate change. The future coffee varieties should be suitably bred to respond appropriately to biotic and abiotic stresses which not only ensure their chances of survival but also affect the yields positively. In this context, it is imperative to identify and functionally characterize the NAC TFs and their promoters in coffee. At present, there is a large paucity of data on coffee transcription factors. Considering the importance of NAC TFs in plant growth, development and defense, further research on NAC TFs in coffee become urgent and indispensable.

## Data availability statement

The datasets presented in this study can be found in online repositories. The names of the repository/repositories and accession number(s) can be found in the article/Supplementary material.

## Author contributions

MM designed the experiment, collected the material, participated in full length gene isolation, project administration and reviewed the manuscript. AH and PJ carried out the experiments and participated in manuscript preparation. SE, GI, RM, and DV participated in *in silico* analysis, review, and manuscript editing. All authors read and approved the published version of manuscript.

## Footnotes

1
https://www.ncbi.nlm.nih.gov/

2
http://www.bioinformatics.nl/cgi-bin/primer3plus/primer3plus.cgi

3
https://www.ncbi.nlm.nih.gov/tools/primer-blast/index.cgi?INPUT_SEQUENCE=%20EU563945.2&LINK_LOC=nuccore

4
https://www.ncbi.nlm.nih.gov/Structure/cdd/wrpsb.cgi
