# Supplementary material for: Comparative genomic analysis and phylogeny of NAC25 gene from cultivated and wild Coffea species
Source: Front Plant Sci. 2022 Sep 16;13:1009733. doi: 10.3389/fpls.2022.1009733 (PMC9523601; doi:10.3389/fpls.2022.1009733)
Supplement: Supplementary file 1 [file Table_5.DOCX]

**Table S1 :** Table depicting Single Nucleotide polymorphism (SNP) resulting Synonymous (S) and Non-synonymous (NS) protein at NAC25 nucleic acid sequence of six coffee species with reference to *C. canephora*.

1. ***C. arabica***

| **Sr. No.** | **Position** | ***C. canephora*** | **SNP** | **S/NS** |  | **Sr. No.** | **Position** | ***C. canephora*** | **SNP** | **S/NS** |
| --- | --- | --- | --- | --- | --- | --- | --- | --- | --- | --- |
| 1 | 447 | C | T | S |  | 4 | 1666 | T | C | NS |
| 2 | 1230 | T | C | NS |  | 5 | 1769 | G | A | NS |
| 3 | 1326 | A | T | NS |  | 6 | 2219 | C | T | NS |

1. ***C. travancorensis***

| **Sr. No.** | **Position** | ***C. canephora*** | **SNP** | **S/NS** |  | **Sr. No.** | **Position** | ***C. canephora*** | **SNP** | **S/NS** |
| --- | --- | --- | --- | --- | --- | --- | --- | --- | --- | --- |
| 1 | 130 | T | C | NS |  | 72 | 1250 | T | A | NS |
| 2 | 131 | T | C | NS |  | 73 | 1252 | C | A | NS |
| 3 | 393 | T | C | S |  | 74 | 1256 | T | G | NS |
| 4 | 418 | A | T | NS |  | 75 | 1296 | C | T | NS |
| 5 | 425 | G | C | NS |  | 76 | 1299 | T | C | NS |
| 6 | 426 | T | C | NS |  | 77 | 1307 | T | C | NS |
| 7 | 445 | T | C | NS |  | 78 | 1313 | T | A | NS |
| 8 | 447 | C | T | NS |  | 79 | 1346 | A | T | NS |
| 9 | 452 | T | C | NS |  | 80 | 1411 | C | T | NS |
| 10 | 457 | C | T | NS |  | 81 | 1414 | G | A | NS |
| 11 | 471 | T | A | NS |  | 82 | 1717 | A | C | NS |
| 12 | 472 | T | A | NS |  | 83 | 1726 | T | G | S |
| 13 | 474 | T | A | NS |  | 84 | 1728 | A | C | NS |
| 14 | 475 | C | A | NS |  | 85 | 1748 | T | C | NS |
| 15 | 490 | T | A | NS |  | 86 | 1759 | C | A | NS |
| 16 | 493 | G | A | NS |  | 87 | 1760 | G | T | NS |
| 17 | 494 | C | A | NS |  | 88 | 1772 | T | G | NS |
| 18 | 501 | A | T | NS |  | 89 | 1777 | A | T | NS |
| 19 | 502 | C | T | NS |  | 90 | 1782 | T | G | NS |
| 20 | 505 | T | G | NS |  | 91 | 1808 | C | T | NS |
| 21 | 510 | T | A | NS |  | 92 | 1817 | C | T | NS |
| 22 | 514 | T | A | S |  | 93 | 1822 | A | G | NS |
| 23 | 517 | C | T | NS |  | 94 | 1829 | T | G | NS |
| 24 | 524 | A | T | NS |  | 95 | 1833 | T | A | NS |
| 25 | 527 | C | A | NS |  | 96 | 1840 | T | C | NS |
| 26 | 549 | G | C | NS |  | 97 | 1847 | G | A | NS |
| 27 | 550 | T | A | NS |  | 98 | 1854 | A | C | NS |
| 28 | 555 | C | G | NS |  | 99 | 1862 | T | A | NS |
| 29 | 558 | A | C | NS |  | 100 | 1874 | C | T | NS |
| 30 | 565 | G | A | NS |  | 101 | 1896 | G | A | NS |
| 31 | 570 | T | A | NS |  | 102 | 1914 | A | C | NS |
| 32 | 573 | G | C | NS |  | 103 | 1922 | C | G | NS |
| 33 | 580 | A | G | NS |  | 104 | 1930 | G | A | NS |
| 34 | 604 | C | T | NS |  | 105 | 1948 | T | C | NS |
| 35 | 605 | T | G | NS |  | 106 | 1975 | A | G | NS |
| 36 | 614 | G | C | NS |  | 107 | 1979 | G | C | S |
| 37 | 622 | G | T | NS |  | 108 | 2039 | C | T | NS |
| 38 | 629 | C | T | NS |  | 109 | 2086 | C | T | NS |
| 39 | 648 | G | A | NS |  | 110 | 2128 | T | C | NS |
| 40 | 651 | T | A | NS |  | 111 | 2142 | G | C | NS |
| 41 | 652 | T | G | NS |  | 112 | 2150 | C | T | NS |
| 42 | 653 | T | G | NS |  | 113 | 2235 | T | G | NS |
| 43 | 654 | C | T | NS |  | 114 | 2236 | C | A | NS |
| 44 | 656 | G | T | NS |  | 115 | 2239 | C | T | NS |
| 45 | 659 | T | A | NS |  | 116 | 2245 | T | C | NS |
| 46 | 660 | T | G | NS |  | 117 | 2258 | T | G | NS |
| 47 | 661 | A | G | NS |  | 118 | 2295 | A | C | NS |
| 48 | 662 | A | G | NS |  | 119 | 2312 | A | T | NS |
| 49 | 666 | A | T | NS |  | 120 | 2333 | T | A | NS |
| 50 | 667 | A | T | NS |  | 121 | 2358 | G | C | NS |
| 51 | 668 | G | A | NS |  | 122 | 2376 | T | A | NS |
| 52 | 669 | T | G | NS |  | 123 | 2390 | G | T | NS |
| 53 | 885 | T | C | S |  | 124 | 2415 | A | G | NS |
| 54 | 898 | A | G | NS |  | 125 | 2419 | A | G | NS |
| 55 | 899 | G | A | NS |  | 126 | 2451 | C | A | S |
| 56 | 905 | T | A | S |  | 127 | 2452 | T | C | NS |
| 57 | 962 | T | A | NS |  | 128 | 2454 | T | C | NS |
| 58 | 989 | A | T | NS |  | 129 | 2457 | T | C | S |
| 59 | 1004 | C | T | NS |  | 130 | 2459 | G | C | NS |
| 60 | 1027 | T | C | NS |  | 131 | 2461 | A | C | NS |
| 61 | 1071 | C | T | NS |  | 132 | 2464 | G | C | NS |
| 62 | 1090 | T | G | NS |  | 133 | 2465 | C | T | NS |
| 63 | 1110 | A | G | NS |  | 134 | 2466 | T | G | NS |
| 64 | 1130 | T | C | NS |  | 135 | 2467 | T | G | NS |
| 65 | 1160 | A | C | S |  | 136 | 2468 | T | C | NS |
| 66 | 1162 | C | G | NS |  | 137 | 2471 | A | T | NS |
| 67 | 1225 | T | G | NS |  | 138 | 2472 | A | T | NS |
| 68 | 1239 | G | A | NS |  | 139 | 2473 | G | C | NS |
| 69 | 1241 | G | A | NS |  | 140 | 2474 | T | C | NS |
| 70 | 1247 | T | A | NS |  | 141 | 2476 | C | T | NS |
| 71 | 1248 | C | G | NS |  | 142 | 2477 | T | C | S |

1. ***C. bengalensis***

| **Sr. No.** | **Position** | ***C. canephora*** | **SNP** | **S/NS** |  | **Sr. No.** | **Position** | ***C. canephora*** | **SNP** | **S/NS** |
| --- | --- | --- | --- | --- | --- | --- | --- | --- | --- | --- |
| 1 | 447 | C | T | S |  | 23 | 1336 | A | T | NS |
| 2 | 827 | T | C | NS |  | 24 | 1338 | C | T | NS |
| 3 | 886 | T | A | NS |  | 25 | 1347 | C | G | NS |
| 4 | 943 | T | A | S |  | 26 | 1630 | G | A | NS |
| 5 | 984 | T | C | NS |  | 27 | 1640 | A | G | NS |
| 6 | 985 | C | T | NS |  | 28 | 1645 | A | C | NS |
| 7 | 1026 | A | G | NS |  | 29 | 2118 | T | C | NS |
| 8 | 1052 | C | T | NS |  | 30 | 2140 | C | T | NS |
| 9 | 1071 | T | G | NS |  | 31 | 2225 | T | G | NS |
| 10 | 1115 | T | C | NS |  | 32 | 2229 | C | T | NS |
| 11 | 1141 | A | C | NS |  | 33 | 2235 | T | C | NS |
| 12 | 1143 | C | G | NS |  | 34 | 2248 | T | G | NS |
| 13 | 1229 | T | G | NS |  | 35 | 2302 | A | T | NS |
| 14 | 1233 | C | G | NS |  | 36 | 2323 | T | A | NS |
| 15 | 1237 | C | A | NS |  | 37 | 2348 | G | C | NS |
| 16 | 1241 | T | G | NS |  | 38 | 2366 | T | A | NS |
| 17 | 1254 | A | C | S |  | 39 | 2380 | G | T | NS |
| 18 | 1261 | G | A | NS |  | 40 | 2399 | G | A | NS |
| 19 | 1295 | T | C | S |  | 41 | 2405 | A | G | NS |
| 20 | 1301 | T | A | NS |  | 42 | 2409 | A | G | NS |
| 21 | 1305 | A | G | NS |  | 43 | 2416 | G | A | NS |
| 22 | 1329 | A | G | NS |  |  |  |  |  |  |

1. ***C. wightiana***

| **Sr. No.** | **Position** | ***C. canephora*** | **SNP** | **S/NS** |  | **Sr. No.** | **Position** | ***C. canephora*** | **SNP** | **S/NS** |
| --- | --- | --- | --- | --- | --- | --- | --- | --- | --- | --- |
| 1 | 130 | T | C | S |  | 40 | 1896 | T | G | NS |
| 2 | 447 | C | T | S |  | 41 | 1897 | T | A | NS |
| 3 | 458 | C | T | NS |  | 42 | 1925 | C | T | NS |
| 4 | 485 | C | A | NS |  | 43 | 1926 | A | G | NS |
| 5 | 544 | C | T | NS |  | 44 | 1931 | T | A | NS |
| 6 | 827 | T | C | NS |  | 45 | 1932 | G | C | NS |
| 7 | 886 | T | A | NS |  | 46 | 1933 | G | C | NS |
| 8 | 943 | T | A | S |  | 47 | 1935 | A | G | NS |
| 9 | 984 | T | C | NS |  | 48 | 1936 | A | T | NS |
| 10 | 985 | C | T | NS |  | 49 | 1939 | C | A | NS |
| 11 | 1026 | A | G | NS |  | 50 | 1940 | T | A | NS |
| 12 | 1052 | C | T | NS |  | 51 | 1941 | G | C | NS |
| 13 | 1071 | T | G | NS |  | 52 | 1951 | T | C | NS |
| 14 | 1115 | T | C | NS |  | 53 | 1978 | A | G | NS |
| 15 | 1141 | A | C | NS |  | 54 | 1982 | G | C | NS |
| 16 | 1143 | C | G | NS |  | 55 | 2040 | C | T | NS |
| 17 | 1204 | T | G | NS |  | 56 | 2056 | C | A | NS |
| 18 | 1205 | T | C | NS |  | 57 | 2072 | A | G | NS |
| 19 | 1210 | T | G | NS |  | 58 | 2085 | T | C | NS |
| 20 | 1224 | T | G | NS |  | 59 | 2089 | C | T | NS |
| 21 | 1228 | C | G | NS |  | 60 | 2131 | T | C | NS |
| 22 | 1232 | C | A | NS |  | 61 | 2153 | C | T | NS |
| 23 | 1236 | T | G | NS |  | 62 | 2238 | T | G | NS |
| 24 | 1249 | A | C | S |  | 63 | 2242 | C | T | NS |
| 25 | 1256 | G | A | NS |  | 64 | 2248 | T | C | NS |
| 26 | 1290 | T | C | S |  | 65 | 2298 | A | C | NS |
| 27 | 1296 | T | A | NS |  | 66 | 2315 | A | G | S |
| 28 | 1300 | A | G | NS |  | 67 | 2336 | T | A | NS |
| 29 | 1324 | A | G | NS |  | 68 | 2361 | G | C | NS |
| 30 | 1331 | A | T | NS |  | 69 | 2379 | T | A | NS |
| 31 | 1333 | C | T | NS |  | 70 | 2393 | G | T | NS |
| 32 | 1342 | C | G | NS |  | 71 | 2412 | G | A | NS |
| 33 | 1671 | T | C | NS |  | 72 | 2418 | A | C | NS |
| 34 | 1808 | G | T | NS |  | 73 | 2419 | C | A | S |
| 35 | 1878 | G | A | NS |  | 74 | 2422 | A | T | S |
| 36 | 1883 | A | T | NS |  | 75 | 2424 | T | C | NS |
| 37 | 1885 | A | G | NS |  | 76 | 2425 | C | G | NS |
| 38 | 1888 | C | A | NS |  | 77 | 2477 | T | C | NS |
| 39 | 1889 | T | A | NS |  |  |  |  |  |  |

1. ***C. jenkinsii***

| **Sr. No.** | **Position** | ***C. canephora*** | **SNP** | **S/NS** |  | **Sr. No.** | **Position** | ***C. canephora*** | **SNP** | **S/NS** |
| --- | --- | --- | --- | --- | --- | --- | --- | --- | --- | --- |
| 1 | 447 | C | T | S |  | 13 | 2102 | C | T | NS |
| 2 | 1298 | A | T | NS |  | 14 | 2191 | C | T | NS |
| 3 | 1638 | T | C | S |  | 15 | 2197 | T | C | NS |
| 4 | 1741 | G | A | NS |  | 16 | 2210 | T | G | NS |
| 5 | 1845 | G | A | NS |  | 17 | 2264 | A | T | NS |
| 6 | 1850 | A | T | NS |  | 18 | 2285 | T | A | S |
| 7 | 1855 | C | A | NS |  | 19 | 2310 | G | C | NS |
| 8 | 1863 | T | G | NS |  | 20 | 2328 | T | A | S |
| 9 | 1888 | C | A | NS |  | 21 | 2342 | G | T | NS |
| 10 | 1899 | C | G | NS |  | 22 | 2367 | A | G | NS |
| 11 | 1904 | C | T | NS |  | 23 | 2371 | A | G | NS |
| 12 | 2080 | T | C | NS |  |  |  |  |  |  |

1. ***C. khasiana***

| **Sr. No.** | **Position** | ***C. canephora*** | **SNP** | **S/NS** |  | **Sr. No.** | **Position** | ***C. canephora*** | **SNP** | **S/NS** |
| --- | --- | --- | --- | --- | --- | --- | --- | --- | --- | --- |
| 1 | 447 | C | T | S |  | 44 | 2235 | C | A | NS |
| 2 | 842 | A | T | NS |  | 45 | 2242 | A | G | NS |
| 3 | 1299 | A | T | NS |  | 46 | 2244 | T | C | NS |
| 4 | 1643 | T | C | NS |  | 47 | 2245 | T | C | NS |
| 5 | 1646 | A | T | NS |  | 48 | 2253 | A | C | NS |
| 6 | 1658 | A | C | NS |  | 49 | 2254 | T | A | NS |
| 7 | 1664 | T | C | NS |  | 50 | 2255 | G | C | NS |
| 8 | 1674 | A | T | NS |  | 51 | 2263 | C | A | NS |
| 9 | 1677 | A | C | NS |  | 52 | 2264 | T | A | NS |
| 10 | 1683 | T | G | NS |  | 53 | 2265 | T | A | NS |
| 11 | 1685 | A | T | NS |  | 54 | 2267 | A | G | NS |
| 12 | 1700 | T | C | NS |  | 55 | 2272 | G | A | NS |
| 13 | 1701 | G | C | NS |  | 56 | 2283 | T | G | NS |
| 14 | 1702 | T | G | NS |  | 57 | 2285 | T | A | NS |
| 15 | 1708 | A | C | NS |  | 58 | 2286 | T | C | NS |
| 16 | 1723 | A | C | NS |  | 59 | 2287 | T | A | NS |
| 17 | 1740 | A | T | NS |  | 60 | 2290 | T | A | NS |
| 18 | 1748 | T | C | S |  | 61 | 2291 | C | T | NS |
| 19 | 1750 | A | T | NS |  | 62 | 2292 | C | T | S |
| 20 | 1761 | C | T | S |  | 63 | 2311 | T | G | NS |
| 21 | 1764 | A | T | NS |  | 64 | 2314 | T | A | NS |
| 22 | 1765 | C | T | NS |  | 65 | 2322 | T | G | NS |
| 23 | 1766 | G | T | NS |  | 66 | 2325 | T | A | NS |
| 24 | 1779 | A | T | NS |  | 67 | 2326 | A | C | NS |
| 25 | 1780 | G | T | NS |  | 68 | 2329 | A | G | NS |
| 26 | 1783 | T | G | NS |  | 69 | 2333 | T | A | S |
| 27 | 1792 | T | C | NS |  | 70 | 2335 | T | C | NS |
| 28 | 1793 | G | A | NS |  | 71 | 2336 | T | A | NS |
| 29 | 1821 | A | T | NS |  | 72 | 2347 | G | T | NS |
| 30 | 1933 | A | T | NS |  | 73 | 2349 | T | A | NS |
| 31 | 1934 | A | C | NS |  | 74 | 2369 | A | G | S |
| 32 | 1935 | A | C | S |  | 75 | 2372 | A | G | NS |
| 33 | 2047 | A | G | NS |  | 76 | 2379 | C | A | NS |
| 34 | 2085 | T | C | NS |  | 77 | 2394 | T | A | NS |
| 35 | 2107 | C | T | NS |  | 78 | 2405 | A | C | S |
| 36 | 2127 | T | C | NS |  | 79 | 2409 | T | C | NS |
| 37 | 2142 | C | T | NS |  | 80 | 2411 | T | C | NS |
| 38 | 2146 | C | T | NS |  | 81 | 2414 | T | C | S |
| 39 | 2149 | C | T | NS |  | 82 | 2416 | G | A | NS |
| 40 | 2193 | C | T | NS |  | 83 | 2417 | C | T | NS |
| 41 | 2196 | C | T | NS |  | 84 | 2422 | C | T | NS |
| 42 | 2199 | T | A | NS |  | 85 | 2430 | G | A | NS |
| 43 | 2208 | T | C | NS |  | 86 | 2431 | T | C | NS |
